# Color-Ratio Maps Enhanced Optical Filter Design and Its Application in Green Pepper Segmentation

**DOI:** 10.3390/s21196437

**Published:** 2021-09-27

**Authors:** Jun Yu, Toru Kurihara, Shu Zhan

**Affiliations:** 1 Graduate School of Engineering, Kochi University of Technology, Kami, Kochi 782-8502, Japan; 218005n@gs.kochi-tech.ac.jp; 2 School of Information, Kochi University of Technology, Kami, Kochi 782-8502, Japan; 3 School of Computer Science and Information, Hefei University of Technology, Hefei 230601, China; shu_zhan@hfut.edu.cn

**Keywords:** optical filter, color-ratio map, green pepper, segmentation, deep learning, precision agriculture

## Abstract

There is a growing demand for developing image sensor systems to aid fruit and vegetable harvesting, and crop growth prediction in precision agriculture. In this paper, we present an end-to-end optimization approach for the simultaneous design of optical filters and green pepper segmentation neural networks. Our optimization method modeled the optical filter as one learnable neural network layer and attached it to the subsequent camera spectral response (CSR) layer and segmentation neural network for green pepper segmentation. We used not only the standard red–green–blue output from the CSR layer but also the color-ratio maps as additional cues in the visible wavelength and to augment the feature maps as the input for segmentation. We evaluated how well our proposed color-ratio maps enhanced optical filter design methods in our collected dataset. We find that our proposed method can yield a better performance than both an optical filter RGB system without color-ratio maps and a raw RGB camera (without an optical filter) system. The proposed learning-based framework can potentially build better image sensor systems for green pepper segmentation.

## 1. Introduction

Improving the quality and production efficiency of the economic crop while aiding the management and marketing strategy is one of the critical aims of precision agriculture. Precision agriculture can provide useful information in the early stage to enable better decision making on the management system. In recent years, computer vision and artificial intelligence technology have developed to meet the growing demand for fast and accurate grain crop production [1,2]. As reviewed by a previous study [3], machine learning techniques have been widely used for the early and precise detection of biotic stress in the crop, specifically for the detection of weeds, plant diseases, and insect pests.

Green pepper is one of the chief crops in Kochi Prefecture, which contributes to approximately 11% of the total production in Japan. Therefore, there is a significant need for using the latest precision agricultural technology to improve the production efficiency of green pepper. Developing automated green pepper harvest and growth prediction technology is essential for farmers to enhance their carriage efficiency and to aid their marketing strategies. However, due to the green peppers and its leaves having the same color, there remains a need for developing robust methods to recognize and segment green pepper. Recently, a new sensor system [4] for the detection and localization of green pepper has been proposed by utilizing multiple camera positions and viewing angles. Li et al. [5] proposed a novel pose estimation algorithm for sweet pepper.

**Figure 1:**
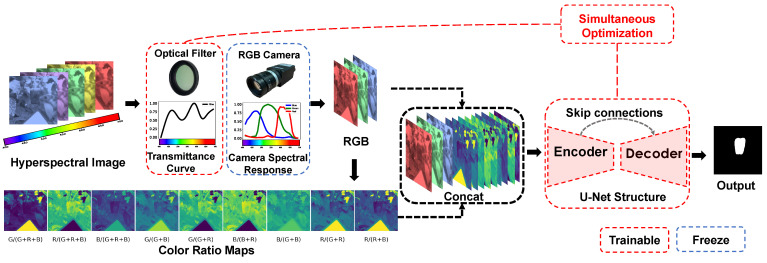
Our proposed computational optics framework incorporates both optics and image segmentation algorithm designs. Rather than optimizing these two parts separately and sequentially, the whole framework was treated as one neural network and establishes a simultaneous end-to-end optimization framework. Explicitly, the first layer of the network corresponds to the physical optical filter, the second layer of the network is related to RGB camera spectral response, and all subsequent layers represent the segmentation algorithm. Inspired by previous research, instead of generating red–green–blue (RGB) channels for the segmentation module, we augment the RGB channels using color-ratio maps to exploit useful spectral information for green pepper segmentation. All of the parameters of the framework are optimized based on segmentation loss on our hyperspectral dataset. Once the transmittance curve is optimized, we can fabricate the corresponding optical filter using multilayer thin-film technology. The fabricated optical filter is mounted in front of the camera lens, and the optimized segmentation network is integrated with the whole system.

Instead of independently optimizing the optical device and relevant image segmentation algorithm, we proposed the optical filter designing method for segmentation neural networks in which the input is enhanced by color-ratio maps. The transmittance curve (TR curve) of the optical filter can be treated as the weight of the neural network, and we can simultaneously optimize both an optical element and green pepper segmentation module by back-propagation. We illustrate the overview of our proposed method in Figure 1. Recently, Yu et al. [6] proposed an end-to-end deep learning optimization algorithm to search for the optimal TR curve of an optical filter in the smooth and non-negative space. However, their method did not fully utilize all of the color-ratio maps from the R, G, and B channels captured by the RGB camera. Historically, agricultural studies investigating color ratios and their linear combinations have shown the effectiveness of distinguishing between fruits and vegetables [7].

In this study, we enhanced the method by adopting the color-ratio maps as input for the segmentation neural network. Color is an important clue of an object’s surface properties. The benefit of color-ratio maps is that it can help us to retrieve the adequate ratio of three chromatics in chromaticity space to derive the optimal TR curve for a specific CSR. In our segmentation module, a U-Net-like structure network [8] is utilized for extracting the spatial features of the RGB images captured by the optimal TR curve of our designed optical filter. After optimization, the designed optical filter can be implemented by optical technology and is attached in front of the camera lens. The spectral property of the incident light is changed by our designed optical filter.

The main contributions of our study conclude as follows:

- We developed the computational optics framework for co-design of an optical filter and segmentation algorithm that can achieve a better image sensor system for green pepper segmentation. The whole framework simultaneously optimizes the front-end optical device (optical filter) and the back-end green pepper segmentation algorithm.
- We introduced the color-ratio maps as additional input feature maps to improve the green pepper segmentation results. The experimental results demonstrate the benefits of the improved performance by color-ratio maps.

The rest of this paper is organized as follows. Section 2 presents the research works related to our work. Section 3 presents the details of our proposed methods. Section 4 describes our green pepper dataset and experimental results. Lastly, Section 5 conclude our presented work and our future work.

## 2. Related Work

### 2.1. Color Space

Color space is the fundamental research topic in color image processing and has various computer vision applications. One of the major current focuses in the advanced driving assistant system is to find an appropriate color space for the detection of traffic lights. In their study, various color spaces were applied for their deep learning model, and the experimental results showed that the RGB and normalized RGB color spaces [9] achieved the best performance. In an earlier study, Kondo et al. [7] established utilizing the color-ratio map to search the most suitable wavelength to distinguish fruits and leaves. In precision agriculture, Zhao et al. [10] proposed using an adaptive RB chromatic aberration map (ARB) based on an OHTA color space [11] and the sum of absolute transformed difference feature in RGB camera to detect immature green citrus. Recently, a novel global image enhancement method, Neural Curve layers [12], was developed by exploiting global image adjustment curves in three different colors spaces, e.g., CIELLab, HSV, and RGB.

### 2.2. Application of Optical Filter

The color filter array (CFA) or multispectral filter array [13] plays an essential role in acquiring the color information or spectral information in the RGB camera and multispectral camera. One of the early and intuitive studies of the optical filter is filter-wheel camera [14]. A series of special optical filters are installed in the rotating filter wheel, where each optical filter can be placed in the optical path of a monochrome camera by rotating the filter wheel. A complete multispectral image is constructed by multiple exposures for different optical filters at a time. Inspired by the CFA in the RGB camera, a multispectral filter array approach was proposed in both academic and industrial areas [15]. Lapray et al. [16] reported a detailed study of the snapshot multispectral imaging and the analysis of spectral filter array. In the real application, Nakauchi [17] proposed a data-driven selection algorithm of a set of bandpass optical filters for ice detection using hyperspectral imaging. They implemented their proposed optical filter by installing two bandpass filters with a near-infrared camera. Another important application of spectral optical filter array is skin oxygenation measurement for medical monitor and diagnosis [18]. Recently, Ono proposed an innovative multi-spectral imaging system using a polarization camera that captures nine bands at once [19].

### 2.3. Computational Optics

Computational optics, which can be interpreted as jointly optimization optics elements (i.e., Bayer color filter, lenses, and optical filters), image processing, and computer vision task, have generated considerable research interest [20,21]. Chang et al. [22] proposed the end-to-end optimization paradigm by combining a differentiable optical image formation layer and a depth estimation network for jointly optimizing both camera lens and neural network weights. Inspired by the recent deep optics approach, A. Metzler et al. [23] developed an end-to-end method to jointly optimize the point spread function of the custom diffractive optical element (DOE) and the deep neural network for high-dynamic-range imaging. Nie et al. [24] reported the relationship between the 1×1 convolution operation and the camera spectral response (CSR) function. They developed a data-driven method to design a camera spectral filter array for hyperspectral reconstruction. Zou et al. [25] proposed the CSR-Net, which can effectively design the optimal CSR to achieve high classification accuracy with limited image bands. A mathematical approach [26] to improve the color measurement of the camera was developed by designing the spectral sensitivity of an optical filter. Their study demonstrated a numerical computation method for optical filter design based on both the Luther condition and the Vora-Value.

## 3. Proposed Method

In this section, we elaborate on our proposed method. We first introduce the filtered RGB camera module. Then, we report the green pepper segmentation module. Lastly, we describe the loss function and physical constraint.

### 3.1. Filtered RGB Camera Module

As illustrated in Figure 1, our proposed filtered RGB camera module consists of two major parts: (1) a differentiable optical filter layer, in which the trained weight is the transmittance curve of the optical filter, that can take in radiance as input and a modified spectral radiance as output; (2) the frozen weights of a convolutional layer with three filters representing the camera spectral response function of the Bayer color filter array.

#### 3.1.1. Optical Filter Layer

Similar to the photographic filter (e.g., UV filter and ND filter), the designed optical filter is mounted directly onto the camera lens. Hence, the spectral information of the incident light at different wavelengths is selectively filtered by the TR curve of the optical filter. We can describe the wavelength-wise product as follows:

(1) L(x,y,λ)=R(x,y,λ)∘T(λ),

where the R(x,y,λ) denotes the radiance data in the captured scenes, T(λ) represents the transmittance curve of the optical filter, and the L(x,y,λ) represents the output radiance data of the designed optical filter, respectively. The ranges of *x* and *y* are 1≤x≤W and 1≤y≤H, where *W* and *H* represent the width and height, respectively, of the captured image in the spatial domain. According to Equation (1), we found the similarity between the depth-wise convolution layer without bias and the TR curve of the optical filter. The depth-wise convolution layer was proposed in the Xeption network structure [27], in which the purpose is to reduce the computation resources. By utilizing the depth-wise convolution layer without bias, the TR curve of the optical filter can be regarded as one layer of the whole neural network structure. One feature of the TR curve is the spatially invariant, i.e., it only works in the spectral dimension and keeps the same transmittance across the spatial dimension in the captured radiance R(x,y,λ). Due to the above feature of the TR curve, we chose the 1×1 as the kernel size of the depth-wise convolution kernel. Each weight in the depth-wise convolution kernel only works on the corresponding wavelength, which selectively transmits the input incident light. Additionally, the filter keeps the same weights across all of the spatial domains.

#### 3.1.2. CSR Layer

Considering the output radiance data L(x,y,λ) at position (x,y), the captured intensity by a fabricated image sensor equipped with CFA is calculated by

(2) $$P_{k}\left(x,y\right)=\int_{\lambda }C_{k}\left(\lambda \right)L\left(x,y,\lambda \right)d\lambda ,\;\;k=R,G,B$$

where λ denotes the wavelength and $C_{k}$ is the corresponded CSR function of the CFA, where *k* denotes the red, green, and blue channels. $P_{k}\left(x,y\right)$ is the pixel intensity of the captured scenes. Essentially, we can discretely formulate the above equation by the following equation:

(3) $$P_{k}\left(x,y\right)=\sum_{i=1}^{N}C_{k}\left(\lambda_{i}\right)L\left(x,y,\lambda_{i}\right),\;\;k=R,G,B$$

where the CSR function is represented by the vector form of $C_{k}\left(\lambda_{i}\right)=\left(C\left(\lambda_{1}\right),C\left(\lambda_{2}\right),C\left(\lambda_{3}\right),\cdots ,C\left(\lambda_{N}\right)\right)$ at different sampled wavelength and *N* represents the total number of the spectral bands. As reported by previous research [24], the CSR function can be represented by three kernels with weights of 1×1 convolutional layer. Consequently, $P_{k}\left(x,y\right)$ can be calculated by the feature map generated by the 1×1 convolution layer with three kernels. In our approach, the simulated image pixel intensity is determined by three factors, i.e., the TR curve of the optical filter, the CSR function of specific CFA, and the radiance of the captured scenes. To account for specular highlights and dark current in the simulated RGB image, we normalized the simulated sensor image as the following Equation (4). The actual values for min and max are determined from the training dataset. We add a small number ϵ to avoid the division by zero in the color-ratio maps introduced in the following subsection. In our experiment, through trial and error, we set ϵ=0.01.

(4) $$P_{k}\left(x,y\right)=\frac{P_{k}\left(x,y\right)-\min}{\max-\min}+\epsilon \;\;k=R,G,B$$

We presume that the camera has a linear response function, which commonly clips the simulated image sensor RGB value to emulate sensor saturation using the following equation.

(5) $$f\left(c\right)=\left\{\begin{array}{cc} 0, & \;\mathrm{if}\;c<0,\\ c, & \;\mathrm{if}\;0\leq c\leq 1,\\ 1, & \;\mathrm{if}\;c>1. \end{array}\right.$$

### 3.2. Color-Ratio Maps

Unlike previous research [6] to utilize only generated RGB images, we augment the simulated RGB image by combing through different color-ratio maps. The simulated RGB sensor images are determined using the three main chromatics: red (R), green (G), and blue (B). Inspired by previous research that the color component ratio could help to distinguish fruits and leaves [7], we utilize the color-ratio maps as additional color cues to help the whole framework search the optimal TR curve of the optical filter. To solve the numerous illumination condition in the green pepper grove, we utilized the normalized RGB color maps [28] in our augment color-ratio map. The normalized RGB color-ratio maps are expressed as $d_{1}$, $d_{2}$, and $d_{3}$. They can be computed by the following equations:

(6) $$\begin{array}{c} d_{1}=\frac{G}{R+G+B}\\ d_{2}=\frac{R}{R+G+B}\\ d_{3}=\frac{B}{R+G+B} \end{array}$$

To efficiently derive the adequate transmittance curve of the optical filter, we applied multiple color-ratio maps. Our proposed multiple color-ratio maps are shown in Equation (7).

(7) $$\left\{\begin{array}{c} d_{4}=\frac{G}{\left(G+B\right)}\\ d_{5}=\frac{G}{\left(G+R\right)}\\ d_{6}=\frac{B}{\left(B+R\right)}\\ d_{7}=\frac{B}{\left(G+B\right)}\\ d_{8}=\frac{R}{\left(G+R\right)}\\ d_{9}=\frac{R}{\left(R+B\right)} \end{array}\right.$$

We extract features from multiple input (RGB + color-ratio maps) to exploit valuable information from different color ratios. Specifically, we concatenate the simulated RGB sensor image and their color-ratio maps as a tensor. We send it to the segmentation module that takes the RGB sensor image with the color-ratio maps as input to generate the final segmentation result.

### 3.3. Segmentation Module

For green pepper segmentation, we attach the segmentation module to our filtered RGB camera module. Note that the principal goal of our research is not to propose the state-of-the-art neural network structure for green pepper segmentation but to explore the relative benefit of color-ratio maps enhancement and co-design optical filter with segmentation module. In particular, we adopt the U-Net-like structure [8] in this work because it is commonly used for pixel-wise estimation (e.g., image segmentation and image-to-image translation) and great generalization performance on various tasks.

Table 1 summarizes the overall structure of the segmentation module; followed by the filtered RGB camera module and the segmentation module accepts tensors of size H×W×12; and lastly, yields the corresponding green pepper segmentation results of size H×W×1. In the encoder part, the basic block is a convolution layer followed by a batch normalization layer [29] and rectified linear unit activation function [30]. We can express the building block in the segmentation module formed as follows: (Conv−BN−ReLU)×2. The spatial size of the feature maps in the encoder part is reduced by the max-pooling layer. In the decoder part, the transposed convolution layer [31] is utilized to increase the spatial size of the feature maps while reducing the number of feature maps. In the end, a 1×1 convolution layer handles the feature maps to generate the final green pepper segmentation map. The skip connection design lets the feature maps in the encoder part directly share with the decoder part to avoid losing essential spatial information. In our experiment, the only difference for the segmentation module in each model is the number of input channels. Unlike the color-ratio map enhancement methods, the model without color-ratio maps only needs three channels of input.

### 3.4. Loss Function and Physical Constraint

As illustrated in Figure 1, we simultaneously optimize the TR curve and the segmentation module via the end-to-end system. The total loss function can be described as

(8) $$L_{total}=L_{bce}+\eta L_{smooth}$$

where $L_{bce}$ denotes the binary cross-entropy loss for green pepper segmentation. It is defined as

(9) $$L_{bce}=-\sum_{\left(x,y\right)}^{\left(H,W\right)}\left[G\left(x,y\right)\log P(x,y)+\left(1-G\left(x,y\right)\right)\log\left(1-P\left(x,y\right)\right)\right]$$

where (x,y) is the pixel coordinates and (H,W) is image size: height and width. G(x,y) and P(x,y) denote the pixel values of the ground truth and the predicted segmentation probability map, respectively.

To aid the physical requirements of the TR curve in the optical filter fabrication, we introduce specific physical constraints for optical filter layer. As the filtered incident light should be positive, all of the weights in the TR curve are nonnegative. Moreover, from the manufacturing perspective, the TR curve of the designed optical filter should avoid arbitrary and sudden variation between adjoining wavelengths. Hence, we proposed the physical constraint that can satisfy nonnegative and smooth features as follows:

(10) $$L_{smooth}={∥\mathrm{GW}∥}_{2}^{2}\;\;s.t.\;\;W\geq 0\;$$

where *G* denotes the second derivative matrix for optical filter layer and *W* represents the weights of the 1×1 depth-wise convolution layer. The parameter η controls the smoothness of the TR curve for the optical filter. Due to the nonnegative property of the optical filter weights, we enforced the nonnegative W≥0 to the depth-wise convolution kernel of optical filter layer in the backward training procedure. In our experimental setting, we verified the different smoothness parameter η, e.g., η=0.1, η=0.01, and η=0.001. By explicitly modeling the TR curve of the optical filter with the specific physical constraint, our proposed optical filter layer can represent the property of a physical device in the real world. The actual optical filter is fabricated to have the same transmittance curve as the learned weights in a further study.

## 4. Experimental Results and Analysis

To clearly explain our proposed method and to determine the suitable parameters in the design space, we conduct several experiments and reports in this section. In this section, we report the details of our experimental results and identify the essential parts that contribute to the overall system.

### 4.1. Hyperspectral Dataset

**Figure 2:**
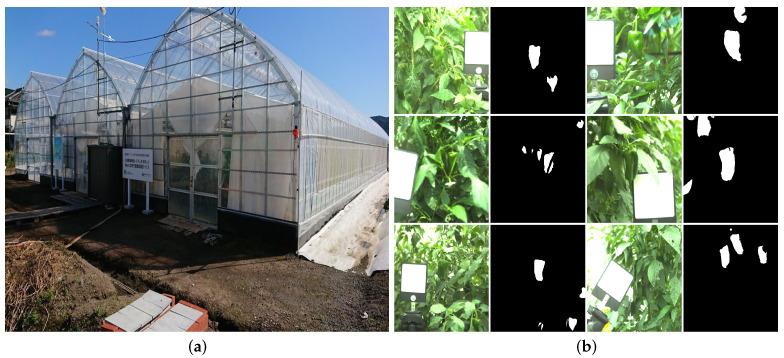
A photograph of the next-generation green house in Kochi University of Technology (KUT), and a sample sRGB image and the ground truth. (**a**) The next-generation green house in KUT. (**b**) Sample sRGB image and ground truth.

Up until now, there has been no public green pepper dataset in the research community and on the Internet. Consequently, to construct a green pepper dataset for our research, we collected hyperspectral images at Next Generation Green House of the Kochi University of Technology and Kochi Agriculture Center, Kochi Prefecture, Japan. We selected a portable push-broom hyperspectral camera (Specim IQ, Specim Ltd., Oulu, Finland) [32] as our data acquisition device. Hyperspectral images of green pepper were collected four times during May 2021 under sunny and cloudy weather conditions. The Specim IQ camera was set to Default Recording Mode(without any processing). In our workflow for image recording progress, we fixed the camera on the tripod and adjusted the camera position and white reference plate position. After that, we manually changed the camera focus and the integration time according to the captured scene. To accurately measure the illumination conditions in the captured scene, we put a standard white reference plate next to our target green pepper in the camera field of view. Sample images are illustrated in Figure 2.

Our hyperspectral camera can record 512×512 pixels image, with 204 spectral bands ranging from 400 nm to 1000 nm. The recording time of our hyperspectral camera for one image is from 40 s to 2 min in the different captured scenes. Compared with the laboratory illumination setting, natural illumination is always inconstant. On the one hand, various factors can affect the spectral power distribution of the illumination, e.g., climate and solar elevation [33]. On the other hand, mutual reflections between different surfaces, occlusions also lead to illumination variation in the natural environment [34]. In the end, we rendered the hyperspectral image to the sRGB image and made the ground truth for green pepper segmentation using the annotation tool LabelMe [35].

### 4.2. Experimental Settings

As mentioned above, we collected a green pepper hyperspectral dataset in our university green house. In total, we obtained 133 hyperspectral images. We randomly selected 101 images as our training set, 16 images as our validation set and 16 images as our test set. In our experiment, we apply the random crop 256×256 image patch from the original hyperspectral image and random horizontal flip to augment the dataset size of our training dataset. As a result, we obtained 7116 training patches for our experiment. Due to the spectral response of our camera being in the visible wavelength, we only used the hyperspectral image from 400 nm to 700 nm. Our experiment was conducted on an NVIDIA Tesla V100 GPU (NVIDIA Corp., Santa Clara, CA, USA) with the deep learning framework PyTorch [36]. The batch size was set to 32. The Adam optimizer [37] with beginning learning rate of 0.001 and β=0.5,β=0.999 was used in our experiment. We dynamic changed the learning rate by monitoring the performance on the validation set. The total epoch was set to 50, and the best model in the validation set was evaluated on the test dataset. The CSR of Lucid Triton 5.0 MP Model [38] was used in our experiment.

### 4.3. Experimental Results

In this section, we compare the performance of different settings of our proposed method with the optical filter (without the color-ratio map) and no optical filter. After that, we illustrate the TR curve of the different settings of our proposed method. Lastly, we evaluate the effectiveness of the color-ratio maps.

#### 4.3.1. Evaluation Results

**Figure 3:**
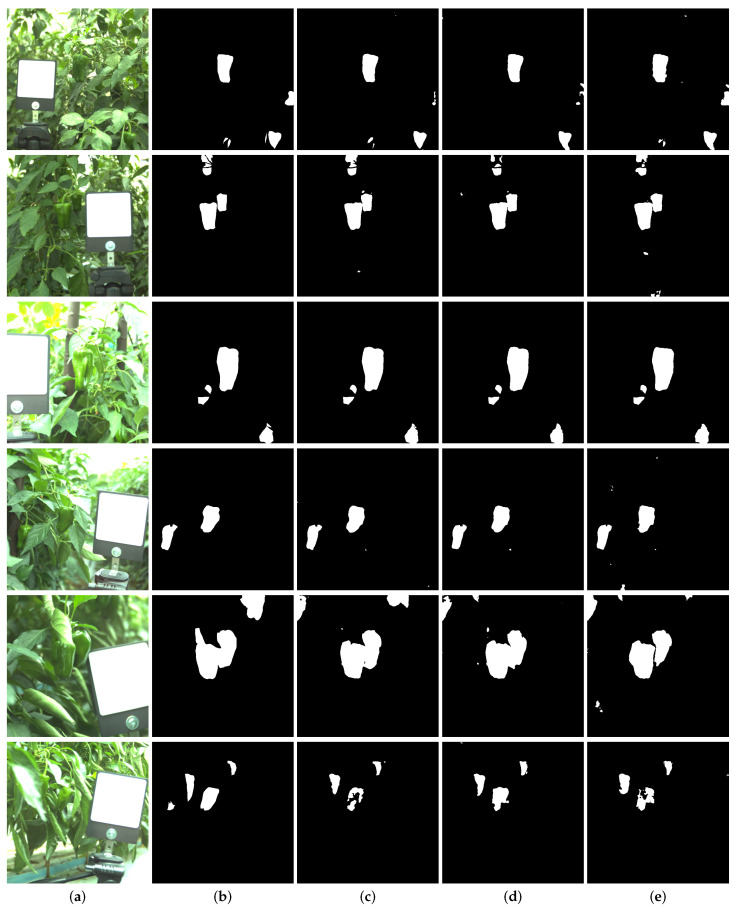
Segmentation results of each model in the test dataset. We only illustrate the best performance of each setting. Each columns show (**a**) Test RGB images. (**b**) Corresponding label. (**c**) The best model in OF-CRM with smoothness η=0.001 and max value max=4.470. (**d**) The best model in OF with smoothness η=0.001 and max value max=1.725. (**e**) The best model in the NF setting with max value max=1.725.

We refer to our proposed method as OF-CRM, that in Yu et al. [6] as OF(Optical Filter), and no optical filter setting as NF(No Filter). To evaluate and compare different setting approaches, we compute the mean intersection over union (mIoU) and F1 measure as the following equations.

(11) $$mIoU=\frac{1}{N_{class}}\sum_{i}\frac{p_{ii}}{t_{i}+\sum_{j}p_{ij}-p_{ii}}$$

(12) $$F_{1}=2\times \frac{precision\times recall}{precision+recall}$$

where $p_{ij}$ is the number of pixels of class *i* predicted to belong to class *j*, there are $N_{\mathrm{class}}$ different classes in total, and $t_{i}=\sum_{j}p_{ij}$ is the total number of pixels of class *i*. In our experiment, the number of classes is set to 2 (pepper or non-pepper). We evaluated different settings of max value in the normalization step, which is used to simulate camera saturation, and smoothness value η, which constraint transmittance curve smoothness. Table 2 shows the results of different maximum values and smoothness η settings. Empirically, we find that the proposed model with η=0.001 and max=4.470 achieves the best performance in all of the settings. We also demonstrate the segmentation results in Figure 3.

**Figure 4:**
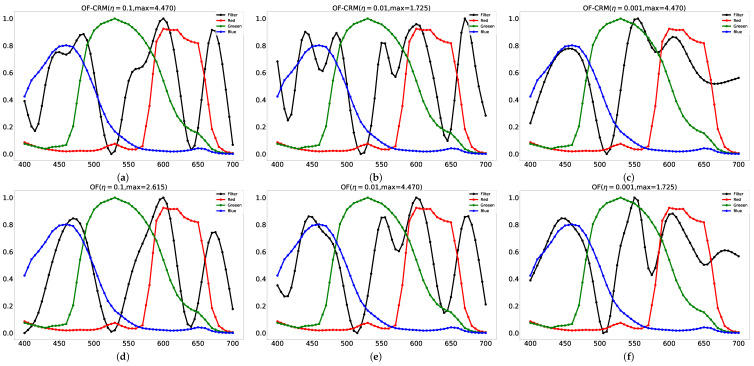
The TR curves of each model is illustrated in the above image. The top row shows the proposed model (with CRM), and the bottom row shows the optical filter design without CRM. In each η setting, we only demonstrate the best model among different max values. (**a**) OF-CRM with smoothness η=0.1 and max value max=4.470. (**b**) OF-CRM with smoothness η=0.01 and max value max=1.725. (**c**) OF-CRM with smoothness η=0.01 and max value max=1.725. (**d**) OF with smoothness η=0.1 and max value max=2.615. (**e**) OF with smoothness η=0.01 and max value max=4.470. (**f**) OF with smoothness η=0.001 and max value max=1.725.

Remarkably, we notice that both the color-ratio maps and smoothness have influenced the shape of the designed TR curve. Looking at Figure 4, it is apparent that the optimal TR curve of η=0.1, η=0.01 turns out to be the multiple bandpass optical filter. Intuitively, there is a clear trend of increasing η when generating more clear bandpass wavelength with the color-ratio map. Upon closer inspection of Figure 4, the transmittance in wavelength around 510 nm and 650 nm is almost zero in all settings, which is not helpful for green pepper segmentation.

In general, most plants look green in our human eyes due to chlorophyll, which is vital for photosynthesis. There are two types of chlorophyll in the land plants, chlorophyll a and b. As reported in the previous study [39], they have different absorption spectrums. For example, the absorption peak of chlorophyll b is just below 650 nm. Interestingly, we can observe that the TR curves of all models are suppressed at wavelengths just below 650 nm. It can thus be suggested that the content of chlorophyll a and b is different in green pepper and leaves. The present study raises the possibility that our optical filter has found these chlorophyll ratio differences in the same green color. This difference appears in the red channel, and it has been proven to play an essential role in distinguishing green pepper and leaves, as we review in the following subsection. However, until now, we have not yet found related studies to support this hypothesis, which supports the chlorophyll ratio difference between green pepper and leaves. A further study with more focus on the ratio of chlorophyll a and b is therefore suggested.

#### 4.3.2. Effectiveness of Color-Ratio Maps

**Figure 5:**
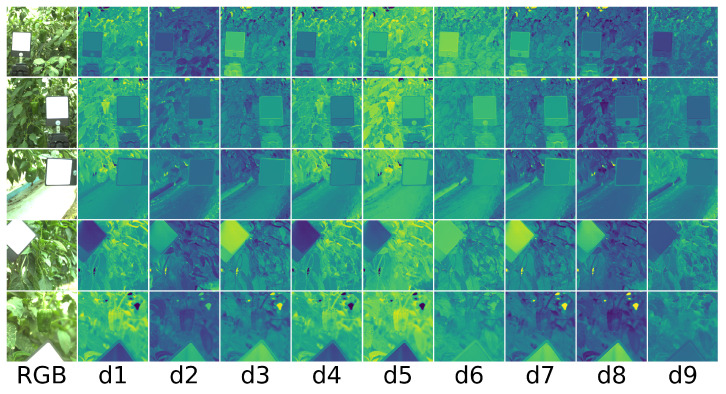
The above figures illustrate color-ratio maps of each test data. The first column shows five different RGB images. The second column to the tenth column shows the different color-ratio maps defined in Equations (6) and (7) for each RGB image in the same row. All color-ratio maps are shown in the same range [0, 1].

To empirically analyze how our proposed color-ratio maps work, we demonstrate the color-ratio maps on the test data. As shown in Figure 5, it is apparent that, in the color-ratio map of d5 and d8, the green pepper is more distinguished. In the color-ratio map d5, the green pepper is highlighted. On the contrary, in d8, the green pepper looks dark than other parts of that color-ratio map. However, some of leaves also look dark, which is similar to the green pepper.

**Figure 6:**
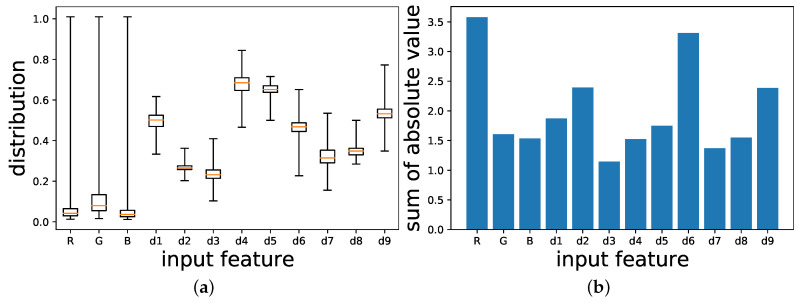
(**a**) The boxplot of the distribution of the input tensor of test dataset for OF-CRM (η=0.001, 4.470). (**b**) Sum of absolute value of all kernels for the input features of a segmentation module in OF-CRM (η=0.001, 4.470). R channel and d2=R/(R+G+B), d6=B/(B+R), and d9=R/(B+R) are more important than the other features and channels.

**Figure 7:**
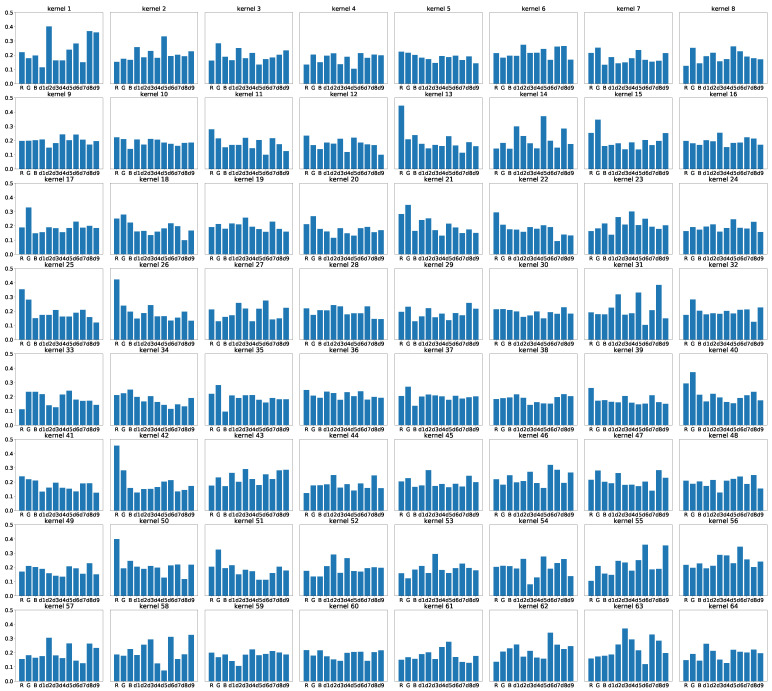
The sum of the absolute values of each input channel for each kernel. The horizontal axis represents the different input features of the segmentation module, R, G, B, d1, d2, d3, d4, d5, d6, d7, d8, and d9 from left to right. The vertical axis of all subfigures are shown in the same range [0, 0.5]. The graph above shows that the color-ratio maps play an essential role, with some kernels showing larger in the color-ratio maps than in the RGB feature map.

To analyze the importance of each input feature, especially color-ratio maps, we adopted the idea of the sum of absolute values of kernels used in filter pruning [40]. In Figure 6a, the distribution of each input feature is demonstrated by the boxplot. As can be seen from the figure, the distribution of the green channel and its corresponding color-ratio maps have slightly larger values than the other channels. However, since the differences are small, we introduced the sum of the absolute values of the kernels into the analysis to interpret the importance of each input feature. Figure 6b illustrates the sum of the absolute values of all kernels for input features, including R, G, B, and all color ratio maps. It seems that the red channel, d2, d6, and d9 are essential inputs for the segmentation module. It turns out that the ratio of the red channel to other channels can provide more meaningful information than other color-ratio maps. A critical hypothesis that emerged from the figure is the red channel is vital for green pepper segmentation. These results provide further support for the hypothesis that the red channel is vital for green pepper segmentation as mentioned in the previous part. We also illustrate the sum of the absolute value of each kernel for each input feature in Figure 7. As we know, each kernel in the convolutional layer pays attention to different input features. The fact that some kernels show large weights for CRM features indicates that the CRM features play an important role in distinguishing green peppers from leaves.

## 5. Conclusions

In this paper, we present an end-to-end optimization approach for the simultaneous design of optical filters and green pepper segmentation neural networks. We aim to leverage an end-to-end deep learning framework to find the optimal TR curve for green pepper segmentation. To accomplish this purpose, we model the critical components inside our end-to-end framework, including the TR curve of the optical filter, CSR of the RGB camera, and our proposed color-ratio maps. Throughout our experiments, we demonstrate the proposed method achieved the best performance in the mIoU and F1 measure.

As opposed to any deep learning-based methods that operate directly on a hyperspectral image or an RGB image, our proposed approach has the ability to optimize both the TR curve of an optical element and the weight of the segmentation module simultaneously. Particularly, the design of TR curve of an optical element is enhanced by the color-ratio maps, which is useful for exploiting the spectral information. This study has been one of the first attempts to thoroughly examine the enhancement of color-ratio maps for optical filter optimization. Our future study fabricates the optical filter according to the designed weights and evaluates its performance in a real application scenario.

## Figures and Tables

**Table 1:** The “U-Net-like”-based segmentation module.

U-Net-Like Encoder			U-Net-Like Decoder		
**Layer**	**Details**	**Size**	**Layer**	**Details**	**Size**
input	R,G,B feature map+ color-ratio map	256 × 256 × 12	upsampling1	2 × 2 upsample of block5 concatenate with block4	32 × 32 × 1024
block1	{conv(3 × 3, pad = 1) + Batch Norm ReLU} × 2	256 × 256 × 64	block6_1	{conv(3 × 3, pad = 1) + Batch Norm ReLU} × 2	32 × 32 × 256
pool1	2 × 2 max pool; stride 2	128 × 128 × 64	upsampling2	2 × 2 upsample of block6 concatenate with block3	64 × 64 × 512
block2	{conv(3 × 3, pad = 1) + Batch Norm ReLU} × 2	128 × 128 × 128	block7	{conv(3 × 3, pad = 1) + Batch Norm ReLU} × 2	64 × 64 × 128
pool2	2 × 2 max pool; stride 2	64 × 64 × 128	upsampling3	2 × 2 upsample of block7 concatenate with block2	128 × 128 × 256
block3	{conv(3 × 3, pad = 1) + Batch Norm ReLU} × 2	64 × 64 × 256	block8	{conv(3 × 3, pad = 1) + Batch Norm ReLU}× 2	128 × 128 × 64
pool3	2 × 2 max pool; stride 2	32 × 32 × 256	upsampling4	2 × 2 upsample of block8 concatenate with block1	256 × 256 × 128
block4	{conv(3 × 3, pad = 1) + Batch Norm ReLU} × 2	32 × 32 × 512	block9	{conv(3 × 3, pad = 1) + Batch Norm ReLU} × 2	256 × 256 × 64
pool4	2 × 2 max pool; stride 2	16 × 16 × 512	outconv	1 × 1 × 1	256 × 256 × 1
block5	{conv(3 × 3, pad = 1) + Batch Norm ReLU} × 2	16 × 16 × 512			

**Table 2:** Quantitative comparison of different models. Our model outperforms the optical filter design without color-ratio maps and no filter settings in the test dataset. The minimum value in Equation (4) is the same in all settings (min = 0.008).

Models	Smoothness	Max	mIoU	F1
OF-CRM	η=0.001	1.725	0.877	0.864
		2.615	0.878	0.874
		4.470	0.899	0.891
	η=0.01	1.725	0.884	0.875
		2.615	0.866	0.853
		4.470	0.887	0.869
	η=0.1	1.725	0.877	0.862
		2.615	0.874	0.862
		4.470	0.877	0.864
OF [6]	η=0.001	1.725	0.875	0.858
		2.615	0.870	0.855
		4.470	0.869	0.846
	η=0.01	1.725	0.850	0.823
		2.615	0.865	0.849
		4.470	0.877	0.862
	η=0.1	1.725	0.864	0.841
		2.615	0.868	0.845
		4.470	0.852	0.822
NF	N/A	1.725	0.867	0.853
		2.615	0.857	0.832
		4.470	0.823	0.815

## Data Availability

Data sharing not applicable.

## Abbreviations

The following abbreviations are used in this manuscript:

CSR	Camera Spectral Response
CFA	Color Filter Array
TR	Transmittance Curve
CRM	Color-Ratio Map
OF	Optical Filter
NF	No Filter
